# Gram-Negative Flagella Glycosylation

**DOI:** 10.3390/ijms15022840

**Published:** 2014-02-19

**Authors:** Susana Merino, Juan M. Tomás

**Affiliations:** Department of Microbiology, Faculty of Biology, University of Barcelona, Diagonal 643, Barcelona 08071, Spain; E-Mail: jtomas@ub.edu

**Keywords:** flagella, Gram-negative, glycosylation

## Abstract

Protein glycosylation had been considered as an eccentricity of a few bacteria. However, through advances in analytical methods and genome sequencing, it is now established that bacteria possess both *N*-linked and *O*-linked glycosylation pathways. Both glycosylation pathways can modify multiple proteins, flagellins from Archaea and Eubacteria being one of these. Flagella *O*-glycosylation has been demonstrated in many polar flagellins from Gram-negative bacteria and in only the Gram-positive genera *Clostridium* and *Listeria*. Furthermore, *O*-glycosylation has also been demonstrated in a limited number of lateral flagellins. In this work, we revised the current advances in flagellar glycosylation from Gram-negative bacteria, focusing on the structural diversity of glycans, the *O*-linked pathway and the biological function of flagella glycosylation.

## 1. Introduction

Protein glycosylation, the covalent attachment of carbohydrates to amino acids, is one of the most common posttranslational modifications in eukaryotic cells. The reaction is performed by glycosyltransferases, which use activated sugar or lipid-phospho sugar donors to drive the reaction in the direction of glycosidic bond synthesis to form a glycan that is covalently attached to a protein. Glycosylated proteins provide mechanisms for the control of signal transduction, protein folding, stability, cell-cell interaction and host immune response. It was considered an exclusively eukaryotic mechanism until the identification of glycoproteins in *Halobacterium* and *Clostridium* [1,2]. Since then, it has become clearly established that several bacterial proteins, especially surface proteins and flagellins are post translationally modified through the linkage to carbohydrates groups [3,4]. Glycans can contain distinctly bacterial sugars, such as the amino- and deoxy-monosaccharides pseudaminic acid [5], bacillosamine [6], 2,4-diacetamido-2,4,6-trideoxyhexose (DATDH) [7], and *N*-acetyl fucosamine [8]. There are two distinct mechanisms to transfer glycans onto a protein acceptor: glycan chains can be assembled on a lipid carrier and transferred *en* bloc onto protein acceptors, by a oligosaccharyltransferases (OTase)-dependent mechanism; or sugars can be attached sequentially onto carrier proteins, by a OTase-independent mechanism. Furthermore, glycans can be linked at several amino acid residues, usually through linkage to the amide group of asparagine residues in *N*-glycosylation or to the hydroxyl group of serine or threonine residues in *O*-glycosylation. Bacterial glycoproteins are usually linked to pathogenesis. Thus, some bacteria with inappropriate flagellar glycans cannot colonize their host [5], while in others, glycans on proteins mediate interaction with the host cells or evasion of the host’s immune system [9,10].

## 2. *N*-Glycosylation

*N*-glycosylation covalently links carbohydrates to asparagine residues in the consensus sequence Asn-X-Ser/Thr (X represents any amino acid except proline) [11]. Although eukaryotes and bacteria can generate *N-*glycans, variety through branching, trimming, lengthening and sugars composition, are larger in bacterial *N-*glycans. *Campylobacter jejunii* was the first bacterium found to have this mechanism [12]. Later on, a growing number of similar protein modification mechanisms have been described in other ɛ- and δ-proteobacteria [4].

*N*-glycosylation includes two evolutionary distinct mechanisms: an OTase-dependent and an OTase-independent mechanism. The *N*-OTase dependent mechanism was typified by the *C. jejuni* PglB system whose glycosylation machinery is encoded by a single locus named *pgl* (protein glycosylation locus), and is responsible for the glycosylation of at least 30 different proteins [6,12,13]. The sequential action of PglF, PglE and PglD catalyze the biosynthesis of UDP-bacillosamine from UDP-*N*-acetyl glucosamine (*N*-GlcNAc). Bacillosamine is transferred to undecaprenyl pyrophosphate (UPP) by PglC and then, extended with five *N*-acetylgalactosamine (GalNAc) residues by the action of PglA, PglJ and PglH GalNAc transferases. PglI is a branching enzyme which adds glucose in a β1, 3-linkage. The assembled heptasaccharide is flipped by the action of PglK and transferred *en bloc* to the asparagine residue of the acceptor polypeptide in the periplasm by an *N*-OTase named PglB [14,15]. This mechanism has significant similarities with the synthesis of *O*-antigen or *O*-polysaccharide in many Gram-negative bacteria. However, although glycan transfer in bacterial *N*-glycosylation typically occurs in the periplasm and involves a membrane-bound OTase, *N*-glycan transfer to proteins can also proceed in the bacterial cytoplasm via soluble glycosyltransferases by an OTase-independent mechanism. This mechanism is typified by the *Haemophilus influenzae* HMW1C cytosolic glycosyltransferase that directly transfers hexose units from nucleotide-activated sugars to multiple asparagine residues of a high molecular weight adhesin [16].

## 3. *O-*Glycosylation

*O*-glycosylation covalently links glycans to serine or threonine residues by a oligosaccharyltransferase (OTase)-dependent or -independent mechanism, although a general consensus amino acid sequence has not been defined for bacterial *O*-glycosylation.

The *O*-glycosylation OTase-dependent mechanism has been described for glycosylate type IV pilin subunits of *Neisseria gonorrhoeae* and *Neisseria meningitidis* [17], but not in eukaryotes. This mechanism is initiated by a specialized glycosyltransferase that attaches a nucleotide activated monosaccharide to an undecaprenolphosphate (Und-P) lipid carrier on the inner face of the plasma membrane [18]. Afterward, glycosyltransferases attach additional monosaccharides to this first sugar residue on Und-PP. When the glycan is completed, the Und-PP linked glycan is flipped to the periplasm and an OTase transfers the glycan to a selected serine or threonine residue in the acceptor protein. This pathway looks like the *N-*glycosylation process described in *Campylobacter jejuni* and shares some similarities with the *wzy*-dependent pathway of lipopolysaccharide *O*-antigen biosynthesis [14,18].

The *O*-glycosylation OTase-independent mechanism has been described to glycosylate flagellins and non-pilus adhesins, such as autotransporters and two-partner secretion pathway exoproteins [19,20]. This mechanism is performed by glycosyltransferases, which sequentially transfer monosaccharides to an acceptor protein.

## 4. Flagellar Glycan Structures

Flagella *O*-glycosylation has been demonstrated in many Gram-negative bacteria and in only *Clostridium* and *Listeria* of the Gram-positive genera [21–23]. Until now, there is no evidence in the literature that the peritrichous flagellins from *Enterobacteriaceae* are glycosylated; however glycosylation of polar flagellins appears to be the norm rather than the exception. Glycosylation of lateral flagellins has been described in a limited number of cases.

The amino acid sequence alignment indicates that flagellin proteins are well conserved in the *N*- and *C*-terminal regions, which correspond to D0 and D1 domains, and the central variable region forms the outside surface-exposed domains (D2 and D3) in the assembled filament. In general, the sites of flagella *O*-glycosylation are localized to the surface-exposed domains of the flagellin subunits [24]; although these sites do not seem to be related to a certain conserved peptide sequence, there often seems to be a hydrophobic region next to the Ser/Thr residues [5,25].

In many cases, the glycosyltransferases and the sugar biosynthetic pathways required for flagellin modification are encoded in close proximity to the genes encoding the flagellar apparatus.

Bacteria can glycosylate their flagellins with differing amounts of glycan at a varying number of acceptor sites. At the two extremes, *Burkholderia* spp. and *Listeria monocytogenes* glycosylate their flagellins at a single site, whereas *Campylobacter jejuni* flagellin is glycosylated up to 19 times, making it one of the most heavily glycosylated proteins identified to date [15,25,26]. Furthermore, some bacteria utilize a single monosaccharide, such as *Helicobacter pylori*, while others have strain-to-strain glycan heterogeneity ranging from monosaccharides to oligosaccharides, such as *Pseudomonas aeruginosa* [21,25,27]. In certain species, there is glycan heterogeneity between strains, such as *P. aeruginosa*, whereas in others the glycan structures are highly conserved. Thus, LPS biosynthesis and the *O*-glycosylation of *Aeromonas caviae* and *Burkholderia* spp. share some genetic components [28,29] and the flagellin of *Azospirillum brasilense* appears to be modified with three to four subunits of a monomer with the same glycan ratio as its LPS *O*-antigen [30].

### 4.1. *Campylobacter* spp

Campylobacters produces bipolar flagella composed of two structural proteins FlaA and FlaB, which are modified with as many as 19 *O*-linked glycans that can constitute up to 10% of the total weight of the flagellin [25]. Most of the modified residues are restricted to the central domain of the flagellin protein which creates the surface-exposed domain when folded in the filament, and these modifications contribute to the flagellum serospecificity [31–33]. The predominant *O*-glycans are derivatives of pseudaminic acid (PseAc, where Ac represents an acetamido group) and an acetamidino form of legionaminic acid (LegAm, where Am represents acetamidino) which are nine-carbon sugars related to sialic acid [34].

Flagellins from *C. jejuni* strain 81–176 and NCTC 11168, and *Campylobacter coli* VC167 are modified with pseudaminic acid (Pse5Ac7Ac), but modifications of *C. jejuni* 81–176 flagellins also include an acetamidino form of pseudaminic acid (PseAm), as well as minor amounts of Pse5Ac7Ac or PseAm substituted with acetyl, *N*-acetylglutamine (GlnAc) or propionyl (Pr) structures (Pse5Am7Ac, Pse5Ac7Ac8OAc, Pse5Am7Ac8GlnAc and Pse5Pr7Pr; where OAc represents an *O*-acetyl group) [25] (Table 1). However, *C. coli* VC167 and *C. jejuni* NCTC 11168 flagellins are also modified with an acetamidino form of legionaminic acid [36,44]. Minor amounts of *N*-methylacetimidoyl derivative of legionaminic acid, as well as PseAc and LegAm extended by a deoxypentose, were also included in flagellins from *C. coli* VC167 [37], whereas minor amounts of a di-*O*-methylglyceric acid PseAc derivative and a related acetamidino form modify flagellins from *C. jejuni* strain NCTC 11168 [36] (Table 1).

The genes encoding the enzymes for biosynthesis of the glycans, the glycosyltransferases involved in flagellin glycosylation and a family of up to seven homologous motility-associated factor genes (*maf* genes), are located adjacent to the flagellin structural genes, *flaA* and *flaB*, in a region that is one of the more variable regions in the *Campylobacter* chromosome [45–47]. Although the function of *maf* genes is not yet clear, some appear to be involved in flagellin glycosylation. Most strains appear to carry the genes for synthesis of pseudaminic acid (*pse* genes) and an acetamidino form of legionaminic acid (*ptm* genes) [34]. However, in *C. jejuni* 81–176 this region contains only genes involved in the synthesis of PseAc and derivatives of PseAc that include an acetylated form (PseAcOAc), an acetamidino form (PseAm), and a form of PseAm with a glutamic acid moiety attached (PseAmOGln) [25,26,44,48].

### 4.2. *Helicobacter* spp

Flagella of *Helicobacter* spp. are assembled at one or both poles of the cell as either a single flagellum or a bundle of filaments, which in some cases are enveloped in an external sheath [49]. The detailed structural characterization of *H. pylori* flagellin revealed that both the FlaA and FlaB structural proteins were post-translationally modified with pseudaminic acid at seven and ten sites, respectively, located in the central core region of the flagellin [5,26]. In comparison with *Campylobacter*, the flagellar glycosylation in *Helicobacter* displays little heterogeneity and only a single sugar is present, in contrast to the numerous related derivatives found in *Campylobacter* (Table 1) [25,37]. Reconstitution and biochemical characterization of the pseudaminic acid biosynthesis pathway showed that *H. pylori* use a similar set of enzymes to *C. jejuni* [50]. It is proposed that the flagellum sheath of *Helicobacter* prevents recognition of the flagellin or glycan by immunoregulatory elements and decreases the evolutionary pressure for heterogeneity [19].

### 4.3. *Pseudomonas* spp

*Pseudomonas* spp. are ubiquitous in nature and frequently isolated as opportunistic pathogens of both plants and animals. *Pseudomonas aeruginosa* has a single polar flagellum which flagellin is classified as either A-type or B-type based on their molecular mass and reactivity with polyclonal antisera [51,52]. Flagellins of both types are glycosylated and genes involved in carbohydrate synthesis cluster together in genomic islands located in the upstream region of their flagellin gene [53,54]. The A-type flagellin shows *O*-glycans at two sites on the central, surface-exposed region of the flagellin and the glycosyl moiety is linked through a rhamnose residue. The genomic islands of *P. aeruginosa* strains which possess A-type flagellins are polymorphic and depending on the genetic elements present, the glycan can be up to 11 monosaccharides long with a heterogeneous structure composed of pentoses, hexoses, deoxyhexoses, hexuronic acids, and possibly deoxyhexoses with amino and formyl substitutions, as in *P. aeruginosa* PAK, whose genomic island contains a cluster of 14 genes (Table 1) [38,53]. Therefore, glycosylation of A-type flagellins appears to have evolved into a two-stage process whereby initial glycosylation with a single rhamnose monosaccharide is likely common to all A-type strains and in some strains, the rhamnose moiety can be further modified through sequential extension with a heterogeneous glycan [55]. Analysis of *wbpL*, *wbpP* and *wbpO* mutants of *P. aeroginosa* PAK, whose encoded proteins are involved in *O*-antigen lipopolysaccharide biosynthesis, show that a complete *O*-antigen unit is not required before flagellin glycosylation occurs since only the *O*-antigen biosynthetic gene *wbpO* was required for the flagellin glycosylation [56]. The B-type flagellin from *P. aeruginosa* PAO1 possesses two serine residues, located close to each other, that are modified. Each site contains a single l-rhamnose residue to which is linked a unique modification of 209 Da containing a phosphate moiety (Table 1). The nucleotide activated sugar precursor TDP- l-Rha is shared between lipopolysaccharide biosynthesis and flagellar glycan biosynthesis [39,40].

In the phytopathogenic bacteria *Pseudomonas syringae* pv*. tabaci* and *P. syringae* pv. *glycinea*, the flagella were found to be glycosylated at six serine residues on the central domain of the flagellin and the *O*-linked glycan was shown to be composed of a di-, tri- or tetrasaccharide of rhamnose residue capped with one modified 4,6-dideoxy-4-(3-hydroxybutanamido)-2-*O*-methylglucose (viosamine) (Table 1) [41,42].

### 4.4. *Aeromonas* spp

Mesophilic aeromonads are ubiquitous water-borne bacteria, considered opportunistic pathogens of both aquatic and terrestrial animals. In addition, some species cause gastrointestinal and wound infections in healthy humans and, less commonly, septicemia in immunocompromised patients [57]. Mesophilic Aeromonas constitutively express a single polar flagellum, although around 60% of strains most commonly associated with diarrhea [58] also are able to express many lateral flagella when grown in viscous environments or on surfaces [59,60].

Polar flagella of *Aeromonas hydrophila* and *Aeromonas caviae* are complex, being composed of two structural proteins, FlaA and FlaB, which are *O-*glycosylated. In *A. caviae* Sch3N the polar flagellins, FlaA and FlaB, were glycosylated with six to eight pseudaminic acid glycans (Pse5Ac7Ac) in their central region and glycosylation was required for flagellar assembly [59,61]. Genes involved in pseudaminic acid biosynthesis of *A. caviae* Sch3N are mapped in the *O*-antigen biosynthetic cluster, rather than adjacent to flagellin structural genes, and their mutation affects the flagellar biogenesis, as well as the *O*-antigen lipopolysaccharide biosynthesis [28]. Furthermore, this region does not contain any motility-associated factor gene (*maf*) involved in the glycan transfer to the flagellins. Bioinformatic analysis showed that the genome of *A. caviae* contains a single *maf* gene homologue (*maf1*), which is adjacent to polar flagellin structural genes and its mutation only abolishes polar flagella formation, even though this strain of *A. caviae* also has inducible lateral flagella [61].

In *A. hydrophila* AH-3, polar and lateral flagella are *O*-glycosylated, although by different carbohydrates moieties. The lateral flagellin (LafA) was modified at three sites with a single monosaccharide related to a pseudaminic acid derivative which is probably phosphorylated (Table 1). However, the polar flagellin was modified at a maximum of six sites with a heterogeneous glycan comprised of a heptasaccharide of three *N*-acetylhexoasmine (with variable addition of 0–2 phosphate groups and 0–2 methyl groups on each), two hexoses, one pseudaminic acid derivative and one unknown monosaccharides of 102 Da (Pse derivative-Hex-Hex-HexNAc-HexNAc-HexNAc-102 Da) (Table 1). The glycan is linked to the polar flagellins through the pseudaminic acid derivative [35]. This heptasaccharide is unrelated to the O34-antigen LPS characterized in this strain consisting of a tetrasaccharide repeat unit of d-mannose, d-GalNAc, and 6-deoxytalose [62]. The genes involved in pseudaminic acid biosynthesis are mapped near to the polar flagellin structural genes and their mutation abolishes polar and lateral flagella assembly and motility, even though the corresponding flagella structural genes and master regulons are transcribed as the wild-type [35]. However, genes for linking the glycans to the polar or lateral flagellins (*maf*) are adjacently located to each flagellar structural locus and their mutation only abrogates one of these flagella formations [63,64].

### 4.5. *Burkholderia* spp

*Burkholderia pseudomallei* is a Gram-negative bipolar saprophytic rod involved in human and animal melioidosis. However, *Burkholderia thailandensis* is generally not considered to be a human or animal pathogen. The use of gel-based glycoproteomics followed by top-down and bottom-up mass spectrometry showed that the flagellins of both strains were modified with *O*-linked glycan moieties and that the glycans decorating the flagella of each species were different. Analysis of proteins demonstrated show that *B. pseudomallei* flagellin proteins were modified with a glycan with a mass of 291.1 Da, while *B. thailandensis* flagellin protein was modified with related glycans with a mass of 300 or 342.1 Da (Table 1). The 291.1 Da glycan from *B. pseudomallei* contain at least one acetyl group, formic acid and possibly an acetamidino group and ammonia. The 342.1 Da glycan from *B. thailandensis* shows similarity with an acetylated hexuronic acid. Mutagenesis analysis of the lipopolysaccharide *O*-antigen biosynthetic cluster demonstrated that it was involved in flagellar glycosylation and motility in *B. pseudomallei* [29].

### 4.6. Azospirillum brasilense

Bacteria of the genus *Azospirillum* have one polar flagellum when grown in liquid media and additional lateral flagella when grown on solid media. In addition to ensuring motility and chemotaxis [65], the polar flagellum has a significant effect on bacterial adsorption to plant roots [66]. It has been found that the polar flagellin of *Azospirillum brasilense* Sp7 is a glycoprotein with a molecular mass of about 100 kDa [67]. Sugar analyses of the polysaccharide revealed that the glycan chains are represented by a branched tetrasaccharide repeating unit with equal amounts of rhamnose (Rha), fucose (Fuc), galactose (Gal), and *N*-acetylglucosamine (GlcNAc) (Table 1). However, while Gal and GlcNAc have the d-configuration, Rha and Fuc have the l-configuration. This glycan shows the same monosaccharide residue ratio as that observed in the *O*-antigen lipopolysaccharide chain of this strain. These data suggest a close relatedness of the flagellin and LPS glycans of *A. brasilense* Sp7 [30].

### 4.7. Shewanella oneidensis

*Shewanella* spp. are motile by a polar flagellum and as mesophilic aeromonads; some strains possess an additional lateral flagellar system whose assembly is induced in specific conditions [68,69]. The polar flagellum contains two flagellins, except for *S. baltica* OS185 and OS195, and the flagellins’ sizes are remarkably heterogeneous within the genus. *S. oneidensis* polar flagellins are glycosylated and further analysis showed that the major flagellin FlaB contains five serine residues with a series of novel *O*-linked posttranslational modifications. Although the exact composition of glycans are unknown, each contains a constant residue similar to nonulosonic acids (neuraminic, legionaminic and pseudaminic acids) and a second residue whose mass varies by 14 Da, presumably due to varying degrees of methylation (Table 1) [43]. The synthesis of the flagellar glycans starts with homologues to PseB and PseC enzymes, which are the first two enzymes of the Pse pathway, and the mutation of PseB homologue leads to a nonmotile phenotype [70]. Analysis of the *S. oneidensis* genome does not show homologues of any other components of the Pse pathway. This data, together with the absence of Pse residues, supports the idea that *S. oneidensis* does not utilize a complete Pse pathway for glycosylation of flagellins, although the modification could be a modified Pse [43].

## 5. The *O*-Linked Pathway for Flagella Glycosylation

The bacterial flagellum is a long, thin filament that protrudes from the cell body. It is structurally divided into an external part, constituted by the filament and the hook, and an internal part embedded in the bacterial cell envelope, the so-called basal body. The flagellar assembly starts in the cytoplasmic membrane by insertion of the MS-ring, the stator of the motor and six components of the export apparatus into the membrane. After the export apparatus assembly, the majority of structural subunits are then secreted through the central channel of the MS-ring for incorporation at the tip of the growing structure [71]. The filament is a cylindrical structure constituted by 11 protofilaments formed by multiple copies of one or various flagellins. The *N*-terminal sequence of the flagellin monomers is recognized and the monomers exported through the export apparatus/basal body structure along the central channel of the growing filament to the tip, where they are then incorporated. A capping protein promotes polymerization, maintaining high levels of flagellin at the polymerization site and avoiding its diffusion into the media [71]. Recently, flagellin monomers were described as transiting unfolded through the filament channel to the tip, the interactions between the *N*- and *C*-terminal sequences of different monomers being essential to pull the next monomer into the growing flagellum [72]. The premature interactions of flagellin monomers in the cytoplasm were prevented by its interaction with the cytosolic chaperone FliS, which binds to a *C*-terminal 40 amino acid region [73,74].

Until now, the bacterial flagellin glycoproteins characterized show the sites of *O*-glycosylation localized to the surface-exposed domains of the flagellin protein assembled in the flagellar filament [24]. Some authors suggest that the chaperone-flagellin interaction may prevent glycosylation of the *C*-terminal region [19]. However, it remains to be established whether this is a consequence of the requirement of specific conserved sequences at *N*- and *C*-termini, which are essential for the transit of the flagellin monomers through the filament and critical to its crystallization into the flagellum, or the specific folded state of the protein and the local environment of serine/threonine residues when it comes into contact with the specific glycosyltransferases.

**Figure 1.**
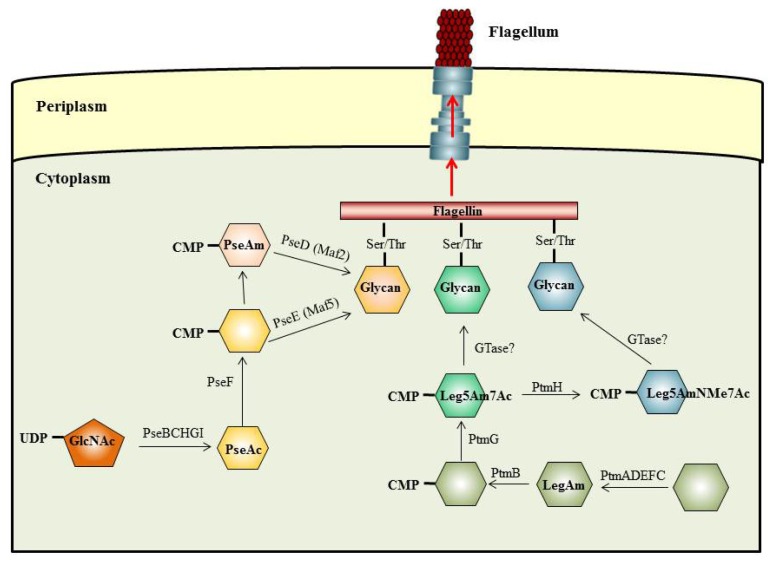
Schematic representation of the hypothetical model for *O*-linked flagellar glycosylation. In Bacteria, *O*-linked flagellin glycosylation is proposed to occur at the cytoplasm-inner membrane interface in the vicinity of the basal body. CMP-activated sugars are sequentially transferring to the Ser or Thr residues in the flagellin monomers by specific glycosyltransferases of the glycosylation machinery (GTase). These glycosylated flagellin monomers are then secreted through the basal body and hook regions to the filament tip, where they are incorporated into the growing filament.

To date, only a few flagellin glycosylation pathways have been elucidated, including the Pse pathway of *H. pylori* and *C. jejuni* [49], the Leg pathway of *C. jejuni* [75], the Wbp pathway of *P. aeruginosa* [56], and the Vio pathway of *P. syringae* [76]. Furthermore, the complete pathway of bacterial flagellin glycosylation is still not clarified although some data suggest that flagellin *O*-glycosylation takes place at the cytoplasm-inner membrane interface in the close vicinity of the basal body by sequential transfer of nucleotide-activated sugar to the serine or threonine residues in the flagellin subunits, as the Figure 1 shows [4,19]. This would permit the assembly of complexes required for the process of glycosylation, in particular for the type of complex glycans composed of multiple monosaccharides. Data supporting that glycosylation occurs in the close vicinity of the basal body and prior to export of flagellin have been found in *C. jejuni* 81–176, since some enzymatic components of the *O*-linked flagellin glycosylation machinery have been localized at the pole of the cell along with the flagella. Furthermore, flagellins *of C. jejuni* 81–176 with a mass that is consistent with full glycosylation could be detected in mutants blocked at early and middle levels of the flagellar hierarchy, as well as in absence of a functional export apparatus [77]. These data, considered together with the transit of unfolded flagellin monomers through the filament channel to the tip, suggest that glycosylation should occur at some point when the unfolded flagellin monomers will be exported by the export apparatus. Furthermore, the modified flagellin monomers should be recognized by the export apparatus prior to export.

## 6. Biological Function of Flagellar Glycans

While the list of bacterial species producing glycosylated flagellins continues to grow, the knowledge of their role in biological systems remains limited for many of them. Many hypotheses for the role of glycans have been proposed, including roles in host mimicry, immune evasion, filament assembly and stability, adhesion and host recognition [4,19]. In general, glycosylation of flagellins is essential for bacterial flagellar assembly, motility, virulence, and host specificity, although in *Pseudomonas* spp. and *Burkholderia* spp., glycosylation is not required for flagellar assembly [15,78].

The flagellins (FlaA and FlaB) of *C. jejuni* and *H. pylori* cannot assemble into filament unless the protein monomers are glycosylated [5,25]. However, the role of specific glycans has been analysed through the use of mutants that decorate flagellins with predominantly one glycan. These studies revealed that PseAm is required for adherence to and invasion of human intestinal epithelial cells as well as for virulence in a ferret model of pathogenesis [48,79,80]. Furthermore, loss of production of LegAm derivatives from a strain of *C. jejuni* lowers the fitness of the bacterium for commensal colonization of chicks [81]. These results suggest that glycan heterogeneity on *C. jejuni* flagellins is required for optimal interaction with various hosts and may play a role in evading certain immune responses [25]. Modifications in the composition of glycans also have consequences in filament stability. Thus, disruption of PseAc biosynthesis *in C. jejuni* or *H. pylori* correlates with reduced levels of flagellins in cell lysates relative to those for wild-type bacteria [5,27,82]. In contrast, *C. coli* which has two independent systems for the biosynthesis of PseAc and LegAm derivatives, the elimination of either system does not affect the levels of flagellins in lysates. However, elimination of both glycan biosynthesis systems severely reduces levels of unsecreted flagellins [82]. Although filament biosynthesis occurs as long as the flagellins are modified with one of the specific *O*-linked glycans, the filament stability is different when it is modified by predominantly one glycan. Thus, in *C. coli* mutants with a filament modified solely by LegAm derivatives, the filament is easily dissociated by SDS, unlike isogenic strains that modify flagellins with solely PseAc or a combination of PseAc and LegAm derivatives [82].

Autoagglutination is another phenotypic characteristic affected by modification in flagellar glycans composition. Thus, in *Campylobacter* the loss of PseAm or PseAc affects autoagglutination although motility was unaffected, suggesting that glycans interact with other flagellar glycans on adjacent bacteria [48]. This phenotypic characteristic likely impacts the ability to form microcolonies and biofilm formation, which is important for interactions with intestinal epithelial cells and other aspects of host colonization [47,81]. Autoagglutination is also affected by the loss of glycans in five of the 19 sites of glycosylation on *Campylobacter* flagellin, while three other sites are critical for flagella assembly and motility [77].

Recent work demonstrates that flagellar hyperglycosylation in *H. pylori* promotes enhanced interaction between bacteria and human gastric epithelial cells, which presumably improves the colonization ability of this bacteria [83].

In contrast to *Campylobacter* spp. and *Helicobacter pylori*, flagellin glycosylation of *P. aeruginosa* was not required for flagellar filament assembly and motility, but a remarkable reduction of virulence was observed when glycosylation was abolished [84]. Furthermore, purified glycosylated flagellin invokes a significantly higher IL-8 immune response whereas the unglycosylated protein display a 50% reduced response [85].

Flagellin from the phytopathogenic bacteria *Pseudomonas syringae* pv*. tabaci* and *P. syringae* pv. *glycinea* has been reported to induce hypersensitive cell death in non-host plants, demonstrating that glycans play an important part in the stimulation of plant hypersensitive response to pathogenic strains [78,86]. Therefore, flagellin glycosylation is involved in host cell recognition and essential for bacterial virulence. On the other hand, flagellar filaments seem to be aggregated into bundles at the cell surface when glycosylation is abolished. This data suggests that glycosylation facilitates proper flagellar suprastructures and lubricates the rotation that contributes to the proper swimming ability of the bacterium, and enhances motility on viscous and sticky surfaces [87,88].

In *Burkholderia* species, as in *Pseudomonas* spp., glycosylation is not required for flagellar assembly and it has been suggested that flagellar glycosylation in *Burkholderia* could be a mechanism used to evade detection by the immune system or to modulate virulence, given the difference in glycan composition and virulence of *B. pseudomallei* and *B. thailandensis* [29].

In mesophilic aeromonads polar and lateral flagellins cannot assemble into filament unless the protein monomers are glycosylated with PseAc. Furthermore, sugar modifications of heptasaccharide that modify the *A. hydrophila* AH-3 polar flagella, as well as the loss of glycan in some glycosylation sites, affects polar flagella stability and motility. Though polar and lateral flagellar systems of the mesophilic Aeromonas are involved in adherence to Hep-2 cells, and surfaces, and in biofilm formation [59,63,64], it is not yet known if glycans play a role in this process.

## 7. Conclusions

Flagellar glycosylation is no longer a rare event found in only a few bacterial species with little biological relevance. Complete genomic information for several bacteria is now available and bioinformatic analysis coupled with functional analysis has allowed the definition of glycosylation pathways and the identification of many genes that participate in flagellin glycosylation. These studies showed that the number of genes involved and their location are diverse in each bacterial species. In spite of these advances, the knowledge of structure and composition of glycans which modify flagellins from Gram-negative bacteria is restricted to certain species, being sometimes strain-dependent. While in some species the sugar involved in flagellin *O*-glycosylation seems to be exclusive, in others, some enzymes are shared with other polysaccharides biosynthetic pathways or the sugars employed are building blocks derived from lipopolysaccharide biosynthesis. On the other hand, many enzymes involved in the transfer of glycans to the flagellin have not been identified or characterized, and the cellular location of this process has not been clarified yet. However, some data suggest that flagellin *O*-glycosylation takes place at the cytoplasm-inner membrane interface in the close vicinity of the basal body.

Functional analyses show that modifications of flagellins with *O*-linked sugars are essential for flagellar assembly in most of glycosylated flagella analyzed. A hypothesis proposes that the *O*-linked glycans facilitate flagellar filament polymerization, but it is not understood how this might occur. In addition, many other functions of flagella glycosylation have been demonstrated, for example filament stability, motility, virulence, gene regulation and mimicry with host-cell surface glycan structure.

Many enzymes involved in bacterial *O*-glycosylation are not present in eukaryotes, and since some of these glycans play a crucial role in pathogenesis, they could be exploited as novel targets for antibiotic and vaccine development, as well as in bacterial infection diagnostics.

## Figures and Tables

**Table 1.** Flagellar glycan structures in Gram-negative bacteria.

Organism	Glycoprotein	Number of sites	Glycan structure	Reference
*Aeromonas caviae* Sch3N	FlaA/FlaB	6/7	Pse5Ac7Ac	[28]
*Aeromonas caviae* UU51	FlaA/FlaB	6/7	Pse5Ac7Ac8Ac	[26]
*Aeromonas hydrophila* AH3	FlaA/FlaB	6FlaB	PseAc derivative-Hex-Hex-HexNAc-HexNAc-HexNAc-102 Da	[35]
	LafA	3	PseAc derivative	[35]
*Azospirillum brasilense* Sp7	Laf1	ND	(→3)-α-l-Rha*p*-(1→3)-β-d-Gal*p*-(1 →3)-β-d-Glc*p*NAc-(1→)*n* = 3–4	[30]
*Burkholderia pseudomallei* K96243	FliC	1	582.4 Da (2 × 291 Da)	[29]
*Burkholderia thailandensis* E264	FliC	1	342 Da	[29]
*Campylobacter jejuni* 81–176	FlaA	19	Pse5Ac7Ac, Pse5Am7Ac, Pse5Ac7Ac8OAc, Pse5Am7Ac8GlnAc, Pse5Pr7Pr	[25,26]
*Campylobacter jejuni* 11168	FlaA	ND	Pse5Ac7Ac, Leg5Am7Ac, Leg5AmNMe7Ac	[36]
*Campylobacter coli* VC167	FlaA	16	Pse5Ac7Ac, PseAm, PseAc/LegAm-deoxypentose, Leg5Ac7Ac, Leg5Am7Ac, Leg5AmNMe7Ac	[37]
*Helicobacter pylori*	FlaA/FlaB	7/10	Pse5Ac7Ac	[5,26]
*Pseudomonas aeruginosa* PAK	FliC	2	11 residues (pentose, hexose, deoxyhexose, hexuronic) attached via l-rhamnose	[38]
*Pseudomonas aeruginosa* JJ692	FliC	2	l-rhamnose [38] *Pseudomonas aeruginosa* PAO1 FliC 2 l-rhamnose and 209 Da phosphate	[39,40]
*Pseudomonas syringae* pv. *tabaci*	FliC	6	β-d-Quip4N(3-hydroxy-1-oxobutyl)2Me-(133)-α-l-Rhap- (132)-α-l-Rhap	[41,42]
*Shewanella oneidensis*	FlaB	5	274 Da and 274 ± 14 Da	[43]

ND: not determined.
